# Managing meat exports considering production technology challenges

**DOI:** 10.1093/af/vfy007

**Published:** 2018-06-15

**Authors:** Haley E Davis, Keith E Belk

**Affiliations:** Center for Meat Safety and Quality, Department of Animal Sciences, Colorado State University, Fort Collins, CO

**Keywords:** beta-agonists, food, hormonal implants, ractopamine, sustainability

**Implications**

- The world population is continuing to grow and could reach as many as 12 billion people in this century, creating a necessity for more sustainable food production.
- Livestock producers utilize production technologies, such as anabolic implants and beta-agonists, to improve sustainability and efficiency of meat production.
- Use of growth promotants in livestock is controversial because several countries will not accept imported meat that is derived from animals that received them.
- Trade barriers associated with use of growth-promoting technologies are widespread, and the countries involved in disputes regarding their use need to find common ground in order to provide high-quality dietary protein to a growing population.

## Introduction

**Figure 1.**
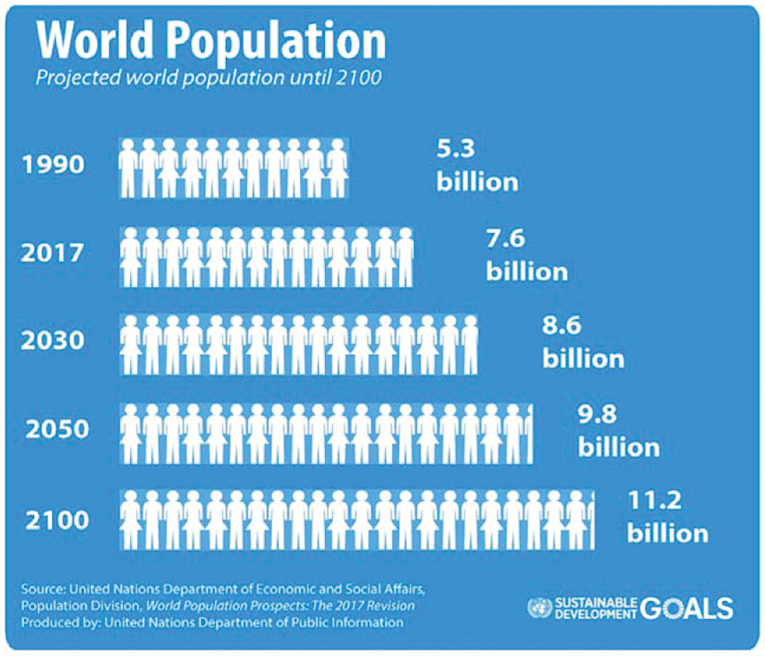
Projected world population from 1990 to 2100.

**Figure 2.**
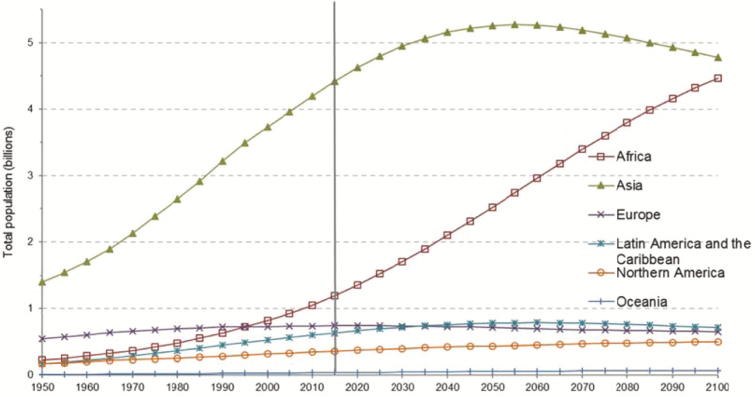
Levels and trends of the world’s population by region. Source: UN-ESA World Population Prospects (2017).

Animal production generating meat, milk, leather, and wool accounts for more than 50% of the value of agricultural products in the United States (USDA-NIFA, 2018). Additionally, livestock products on a global level provide an estimated 13% of total energy and 28% of protein in diets consumed (USDA-NIFA, 2018). Latest population projections by the United Nations indicate that a current global population of 7.6 billion will increase by nearly one billion people in the next 12 yr (UN-ESA, 2017). By 2050, estimates suggest that we will reach a population of 9.8 billion people, and by the new millennia of 2100, we could reach up to an estimated 9.6 to 12.3 billion people (Figs. 1 and 2; Gerland et al., 2014; UN-ESA, 2017). As the global population continues to increase at an alarming rate, so does the necessity to feed more people with fewer resources. Livestock and meat production will be tasked with providing a substantial amount of nutrients and high-quality protein in the future to avoid a calorie deficit globally. For this reason, the livestock industry has relied heavily on technologies, such as anabolic implants and supplementation with beta-adrenergic receptor agonists to aid in increased production efficiencies (Stewart, 2013; Dilger, 2015).

Urbanization and development also impacts animal production as arable land mass continually decreases at a time when there is an ever-increasing need for food. Therefore, production efficiency and sustainability have become major focuses for livestock producers. Overall, the goal of sustainable agriculture is to meet society’s current food and textile needs without compromising the ability of future generations to meet these needs (Kuhlman and Farrington, 2010; Spiertz, 2010). Although the challenge of feeding a growing population is clear, the main concern is whether it can be done sustainably, equitably, and quickly enough to keep up with the growing demand for other resources, such as biofuels (Spiertz, 2010). Agriculturalists have been faced with the dilemma of keeping up with the burgeoning demand for bio-based commodities (food, feed, fiber, and fuel) while also complying with, and satisfying stricter constraints in regard to, product safety and environmental impact (Spiertz, 2010). So, what can be done to face these challenges head on?

Two primary technologies used to more sustainably and efficiently produce livestock and meat are anabolic implants and dietary supplementation with beta-adrenergic receptor agonists, otherwise known as beta-agonists (Johnson et al., 2013). Beef cattle producers have used growth promotants for more than 50 yr, although the technologies have improved over that period of time. These compounds make animals more efficient by increasing ADG of beef cattle with less feed, known as feed efficiency, and thus enhancing the amount of lean muscle produced per unit of inputs (Johnson et al., 2013). Anabolic implants and beta-agonists used in the United States have been embraced by livestock producers due to growth-promoting characteristics which create economic benefits while also allowing for more sustainable animal production (Centner et al., 2014). It is estimated that 90% of agricultural growth, to feed an ever-growing population over the next several decades, must come from more intense production on land currently used for agricultural purposes (Neumeier and Mitloehner, 2013).

Utilization of production technologies, or biotechnologies, has the potential to help in this regard due to improved animal growth, lean yield, and feed efficiency using the same land mass (Neumeier and Mitloehner, 2013). In fact, Stackhouse et al. (2012) demonstrated that use of growth-promoting technologies in feedlot systems in California decreased the carbon footprint, ammonia emissions, and cost of beef production. Beta-agonist use during cattle feeding decreased ammonia emissions, resulting in a 7% decrease in ammonia loss from the full production system (Stackhouse et al., 2012). When a combination of ionophores, hormonal growth implants, and beta-agonists was used, ammonia emissions were further decreased and the carbon footprint was decreased by 2.2 kg carbon dioxide equivalent (Stackhouse et al., 2012). Decreased emissions and improved efficiency improve the overall sustainability of livestock production through generation of more lean protein production (meat and by-products) with fewer inputs, such as grain, water, and land mass (Anderson et al., 2004; Dilger, 2015).

Unfortunately, the shift of consumer preference and political policy positions to natural and organic food production, both in the United States and abroad, has generated trade barriers for products from animals receiving anabolic implants, beta-agonists, and other biotechnologies. For this reason, it is advantageous for politicians, consumers, and producers to better understand these technologies and the challenges associated with them in trade.

### Production Technologies

Use of anabolic steroids, in the form of time-releasing ear implants, has been approved by the United States Food and Drug Administration (**FDA**); they are characterized as safe and effective growth-promoting agents. Hence, producers implant more than 90% of all feedlot cattle in the United States (Johnson et al., 2013; USDA-APHIS, 2013). Since 1954, the FDA has subjected all anabolic implants to strict scrutiny before approval (Stewart, 2013) via the New Animal Drug Application (**NADA**) process. This process requires demonstration and validation that new drugs do not harm people who consume the animal, the animal itself, nor the environment, and that they work as intended (FDA, 2017). Additionally, the FDA uses scientific data to establish acceptable limits for the compounds in meat so that human consumption does not lead to harmful effects (Stewart, 2013; FDA, 2017). The anabolic agents used in beef cattle implants consist of three naturally occurring hormones (estradiol, progesterone, and testosterone) and two synthetic hormones (zeranol and trenbolone acetate [**TBA**]; Stewart, 2013).

These compounds are necessary for normal development, growth, and reproduction of humans and food animals, meaning that people are generally not at risk from consuming food from animals treated with the small quantities that are used to improve efficiency of production with these implants because the additional hormone concentrations present following treatment are miniscule compared to the hormones already generated endogenously in animals and that are normally found in meat products (FDA, 2017). Synthetic hormones must also go through a tedious approval process involving information and/or toxicological testing of laboratory animals to determine safe levels in edible animal products (FDA, 2017).

Implants are small pellets which contain growth promotants that are released gradually over time, thus increasing the circulating levels of somatotropin and insulin-like growth-factor 1 (Stewart, 2013). Growth hormone is then secreted at an accelerated rate, leading to augmented muscle development (Stewart, 2013). Three different hormonal implant strategies exist: androgenic implants, such as TBA and testosterone; estrogenic implants, such as estradiol 17-β (E_2_), estradiol benzoate, and zeranol; and combination implants (androgen plus estrogen), such as TBA plus E_2_ (Johnson et al., 2013). In general, use of hormonal implants has been shown to increase growth rates by up to 28% while improving feed efficiency and lean muscle mass by up to 20% and 10%, respectively (Johnson et al., 2013; Stewart, 2013). Furthermore, there is an additive effect when utilizing combination estrogenic/androgenic implants (Stewart, 2013). In fact, feed efficiency is improved an additional 6% to 14% with combination implants versus estrogen-only implants (Stewart, 2013). It is also estimated that combination TBA/E_2_ implants increase carcass protein by 10% compared to nonimplanted steers, assisting in sustainability of production (Johnson et al., 2013).

Yet another production technology that improves sustainability in livestock production is dietary supplementation with beta-agonists, which are used in feed during the last 3 to 6 wk of finishing (generally for 28–42 d). Beta-agonists are feed additives which are used to improve feed efficiency and promote growth in livestock (Kootstra et al., 2005). Beta-agonists are synthetic compounds which bind to G protein–coupled beta-receptors on cell surfaces in muscle, fat, and other tissues of animals, including humans and livestock (Mersmann, 1998; Johnson, 2014). When beta-agonists bind to adrenergic receptors on cells, they increase muscle mass via hypertrophy, while also decreasing fat accretion/lipid synthesis (Neumeier and Mitloehner, 2013). In other words, beta-agonists lead to increased protein synthesis and decreased muscle protein degradation (Mersmann, 1998) and fat production—hence, some refer to them as “repartitioning agents” because they repartition how nutrients are utilized in metabolism. Beta-agonists are used in human medicine for a number of reasons, such as asthma treatment, but are strictly used as growth promotants in livestock production as they enhance growth and alter body composition (Mersmann, 1998; Anderson et al., 2004). Beta-agonists in livestock production stimulate skeletal muscle growth without an increase in natural hormone levels (Centner et al., 2014). Livestock producers have utilized this technology to increase BW of swine and cattle, eventually leading to heavier carcasses and thus more meat production.

Ractopamine hydrochloride and zilpaterol hydrochloride are the two beta-agonists approved by the FDA for use in food animal species in the United States (Dilger, 2015). Ractopamine is approved for use in swine, turkeys, and cattle and binds to beta-1 receptors, whereas zilpaterol is only approved for use in cattle and binds to beta-2 receptors (Arp et al., 2014; Centner et al., 2014; Dilger, 2015). These beta-agonists are also approved for use in other countries, such as Brazil, Canada, South Korea, and Mexico; however, they have been banned in several places, as well, such as China and the European Union (**EU**) (Dilger, 2015). In the United States, zilpaterol currently is not used in any feeding systems. In a meta-analysis of research data that included more than 50 comparisons for both ractopamine and zilpaterol, dietary supplementation in cattle presented notably increased weight gain, HCW, LM area, and G:F (Lean et al., 2014).

Ractopamine and zilpaterol were both subjected to approval processes similar to the FDA’s approval process for anabolic implants. A NADA begins with the U.S. FDA to ensure safety and effectiveness, and then it is extended to the Center for Veterinary Medicine. From there, the Office of New Animal Drug Evaluation investigates the drug and surveillance and compliance data are accumulated. This process is overseen by FDA veterinarians, animal scientists, biologists, and toxicologists, and takes several years before a decision is made based on scientific evidence. It is a robust system that leaves no credible reason to believe that the drugs that are used are unsafe. According to the makers of livestock growth-promoting technologies, today, the NADA process can take up to 20 yr to result in a newly approved technology, and can cost in excess of $25–$100 million.

Ractopamine, specifically, underwent an extensive approval process through the FDA in order to calculate the no-observed-adverse-effect-level (**NOEL** or **NOAEL**; 0.125 mg/kg/day) and the acceptable daily intake (**ADI**; 1.25 µg/kg/day), which was completed in December of 1999 (FDA, 1999). Its use as a growth promotant was approved in 2000; since then, however, ractopamine use has remained contentious (Centner et al., 2014). After years of scientific review, the Codex Alimentarius Commission, an intergovernmental food standards-setting body with over 180 members, voted to adopt a maximum residue limit (**MRL**) by a narrow vote of 69 to 67 in 2012 (Table 1; Bottemiller, 2012). The Codex Alimentarius Commission considers recommendations from the Joint FAO/WHO Expert Committee on Food Additives and scientific evidence when voting to adopt MRL. This vote made it significantly less difficult for countries with higher tolerances, such as the United States and Canada, to challenge those with zero-tolerance residue policies associated with trade for ractopamine residues in meat products because these policies are more restrictive than the global standard (Bottemiller, 2012). Countries with zero-tolerance policies include China, the EU, and Taiwan (Bottemiller, 2012). In addition, as procedures for importing beef tissues (and particularly beef liver) in Egypt evolve, restrictions in that country are increasing. Even with an international Codex standard, there have been instances in which exports from the United States (and other countries) into countries with zero-tolerance policies were denied due to ractopamine levels that were under the global MRL. And, too, the sample handling and testing methods in such countries can be contentious as they impact results of testing. This controversy remains relevant as ractopamine hydrochloride is still commonly fed to livestock; issues with zilpaterol are less relevant as the compound is not currently used in North America.

**Table 1.** United States and Codex maximum residue limits for ractopamine hydrochloride in regulatory tissues for beef

Tissue	U.S. FDA (ppb)	CODEX (ppb)
Kidney	N/A	90
Liver	90	40
Fat	N/A	10
Muscle	30	10

Notwithstanding the lack of use, zilpaterol also has undergone the NADA approval process; however, maximum residue limits/tolerances have not yet been adopted for this beta-agonist (Arcella et al., 2016) because, when this compound is used, there generally is a 2-d withdrawal from the compound before shipment to packing plants. The Joint FAO/WHO Expert Committee on Food Additives has recommended MRL for zilpaterol in cattle based on several different assessments of the scientific literature, but the Codex Alimentarius Commission has not voted on the issue (Arcella et al., 2016). The recommended limits for cattle are 3.3 µg/kg (or ppm) in kidney, 3.5 µg/kg in liver, and 0.5 µg/kg in muscle (Arcella et al., 2016).

Although zilpaterol was approved as a feed additive for beef cattle in the United States in 2006, several reports of animal welfare concerns arose in the summer of 2013 (Boyd et al., 2015). Consequently, the manufacturer removed zilpaterol from the market as it did not want to contribute to animal welfare problems. Zilpaterol has been linked to increased respiration rates and panting scores in cattle, and also lameness (Grandin, 2014; Boyd et al., 2015). It is important to note that correlation does not equal causation, and research on this topic clearly states that these issues are likely multifactorial (Grandin, 2014; Boyd et al., 2015). Nevertheless, any drug that does, in fact, contribute negatively to animal welfare cannot, and should not, be administered.

Before animal welfare related to beta-agonist feeding became a concern, multiple studies were conducted investigating the additive effects of feeding ractopamine or zilpaterol in conjunction with administration of an anabolic implant. Baxa et al. (2010) investigated effects of zilpaterol hydrochloride and the steroidal implant Revalor-S (a combination implant of TBA and estradiol 17-β) on feedlot performance, carcass characteristics, and skeletal muscle composition in finishing steers. When compared to control cattle, those receiving only zilpaterol and those receiving only the Revalor-S steroidal implant exhibited improvements in growth performance and carcass characteristics (Baxa et al., 2010). As expected, cattle that received the combination treatment presented the greatest increase in average daily G:F, as well as an additive increase in HCW, longissimus multifidus area, and dressing percentage (Baxa et al., 2010). A similar study investigating performance of finishing beef steers in response to anabolic implant and zilpaterol hydrochloride was conducted with differing concentrations of TBA and estradiol (Parr et al., 2011), with similar results of additive responses.

**Figure 3.**
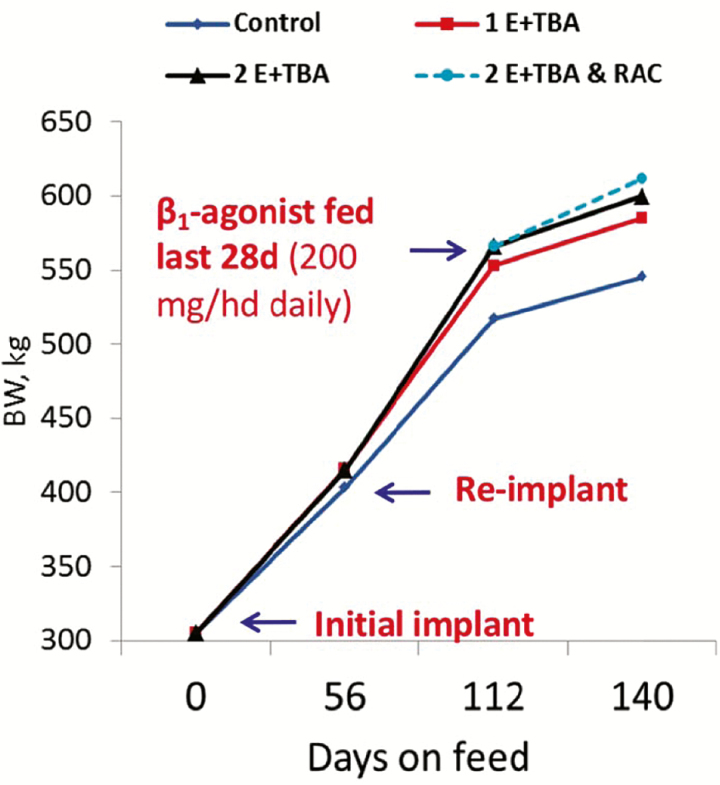
Additive effect of growth enhancement during finishing via steroidal implants and beta-agonists (E + TBA: 17-β estradiol plus trenbolone acetate; RAC = ractopamine hydrochloride). Adapted from Bryant et al., 2010.

In the Parr et al. (2011) study, the higher dose of TBA plus 17-β estradiol with a more gradual release period ameliorated steer performance and HCW (Parr et al., 2011). Bryant et al. (2010) conducted yet another study comparing effects of ractopamine and steroidal implants with differing TBA and 17-β estradiol concentrations in finishing steers. Adapted results from this study are shown in Figure 3. Holding days on feed constant and compared to control cattle, ADG increased by 21% with one anabolic implant; over time, this effect was amplified with a second anabolic implant to nearly 27%, and another 2% with two anabolic implants and dietary administration of ractopamine for the last 28 d of feeding (Bryant et al., 2010). This cumulative and additive effect on growth performance and carcass traits also was observed for G:F (feed efficiency) and carcass weight, with carcass weight increased by more than 100 pounds compared to control cattle (Bryant et al., 2010). These increases alone could lead to an economic benefit of hundreds of dollars, albeit dependent on market values.

Despite the international trade-related controversy surrounding use of beta-agonists as growth promotants, the benefits to sustainability and animal production are obvious. The looming trade issues associated with evolving and ever-better abilities to detect extremely low concentrations of residues in tissues, coupled with unscientific import policies, could impact future ractopamine use.

### Trade Barriers to Exports

Unfortunately, for livestock producers in the United States, various barriers to trade, both tariff and nontariff, adversely affect export markets and create caustic disputes with other countries. One of the barriers results from banning the use of certain growth compounds; China alone lists 146 banned compounds. These banned products include, but are not limited to, anabolic growth-promoting implants and beta-agonists (along with melengestrol acetate and many more compounds), often with a lack of scientific evidence to support the decision-making process.

One of the most hotly contested trade bans in the world resulted from the 1988 European Third-Country Directive that essentially restricted use of natural hormones to strictly therapeutic treatments, while banning utilization of synthetic anabolic agents and the importation of both implant-treated animals and meat from animals to which implants were administered (Johnson, 2015). This ban was implemented despite conclusions published in several reports by a Scientific Working Group of 22 notable European scientists that was formed by the Commission of the European Communities (the forerunner to the EU) and led by Prof. G. E. (Eric) Lamming of the United Kingdom, that clearly refuted any human health consequences of using anabolic growth technologies in livestock production. By 1989, the EU fully implemented this ban on meat and meat products from animals which were administered anabolic growth promotants (Johnson, 2015). This created a major disturbance in meat trade between the United States (along with other countries) and the EU. While there have been many attempts to resolve the issue through World Trade Organization dispute consultations, settlement panels, formal appeals, and arbitration proceedings, it remains problematic to this day as the EU, although losing all attempts to maintain the ban in the World Trade Organization, has retained policies to prevent the use of such growth technologies.

In addition to the ban on use of anabolic compounds, use of beta-agonists as growth promotants in farm animals also was banned in the EU in 1996 (Centner et al., 2014). This ban occurred before the EU conducted any research regarding ractopamine or zilpaterol and prior to beta-agonists entering the market as growth promotants in livestock (Centner et al., 2014). Nearly 10 yr later, after the JECFA reconfirmed the ADI and MRL of ractopamine, the European Food Safety Authority began an investigation to evaluate the compound’s safety because it had not done so before adding it to the list of banned veterinary drugs (Bories et al., 2009). Their investigation considered available information about ractopamine from previous research, including studies examining effects on laboratory animals, dogs, monkeys, pigs, cattle, and humans (Bories et al., 2009). Although no new research was conducted, panelists concluded that the detailed investigation did not provide enough evidence to overturn the ban because it was not clearly stated that the consumption of ractopamine residues by humans was safe (Bories et al., 2009), despite approval by FDA in the United States. Hence, the ban was political, as there was no reason to believe that there was a risk to humans, and therefore a nontariff barrier to trade. Nonetheless, the issue will not dissipate any time soon, and especially as more countries push for zero-tolerance, or other nonscientific protocols, relative to beta-agonist residues.

Trade with China also remains contentious because of banned products, which include zeranol, TBA, and beta-agonists. The Chinese banned products are of significant concern because most North American cattle feeders administer melengestrol acetate as a feed additive to heifers for the purpose of suppressing estrus while increasing BW; this is a banned compound for beef production in China, which completely closes this market to any cattle North American producers that feed heifers.

Ractopamine residues have been in the news frequently since MRL were approved by Codex. Directly after Codex voted to approve the international standards for ractopamine limits, Michael Hansen of Consumers International asserted, “We are concerned that with this vote, Codex is becoming another politicized global body, rather than the science-based consensus body it has managed to be so far” (Bottemiller, 2012). Contrastingly, the chief veterinarian for the National Cattlemen’s Beef Association stated, “It is paramount that science is the foundation for all decisions made in the international community. Today, the Codex commission proved they are willing to trust science and make decisions based on facts rather than politics” (Bottemiller, 2012). Regardless of what side of the issue one is on, it is clear that ractopamine residues have been, and will continue to be, a challenge to the meat industry.

Ractopamine is especially problematic in exported products because tissue concentrations of the compound can be affected by enzymatic reactions that occur during tissue handling. “Parent ractopamine” is the amount of the compound detected in tissues that result directly from the feed additive itself. But, some countries choose to test for “total ractopamine,” which reflects the combination of “parent” ractopamine plus its metabolites. In some postmortem tissues, ractopamine metabolites can undergo enzyme hydrolysis in the presence of beta-glucuronidase to artificially increase the amount of parent ractopamine detected; the consequence can be additional and misleadingly high parent ractopamine concentrations in tissue—perhaps leading to rejection by the importing country. The amount by which tissue concentrations of parent ractopamine are increased is dependent on the tissue, the amount of metabolites present, and the amount of beta-glucuronidase that is in the tissue, along with the temperature at which tissue samples are maintained (higher temperatures result in more rapid conversion of metabolites to parent compound); liver has especially high concentrations of beta-glucuronidase. As an example of how this may result in export problems, consider a customs port in Egypt (which is very warm) where over 80% of livers from the United States are shipped; sample handling in Egypt is critical to export success if inspectors test for parent ractopamine concentrations.

In addition, MRL set by Codex and the FDA are based on concentrations of parent ractopamine levels rather than total levels in the target tissues on which the NADA was based (mainly, liver and muscle). The main concern for producers in the United States who are exporting to countries with limits lower than the regulatory requirements is that the tests used are often specifically for total ractopamine, which results in an escalated quantifiable ractopamine residue that is misleading, and they may be testing off-target tissues to which the MRLs should actually not apply.

Moreover, cross-contamination potential for compounds that are provided to animals in feed is not uncommon in feedlots or at processing plants. In other scenarios, unexpected results of testing frequently occur. Zeranol, a naturally occurring estrogen-like mycotoxin, can be detected in cattle (and particularly in their livers) that have never received a zeranol anabolic implant treatment (i.e., Ralgro) because zearalenone can be generated by certain *Fusarium* species in grains that are fed to the cattle (Kennedy, 1998). At processing plants, further contamination of hormones and beta-agonists can occur when residual compounds are transmitted from gastrointestinal tissues onto processing equipment, leading to positive tests on subsequently cross-contaminated tissues that are not necessarily representative of the actual residual amount.

## Conclusions

Use of growth technologies has helped the livestock and meat industries to make great strides toward sustainability and efficiency of production. It is necessary that such technologies continue to be used if we are to provide high-quality, nutrient-dense proteins to a rapidly growing global human population. Regrettably, not all policies (and, particularly, import policies) around the globe agree about the appropriateness of use for growth technologies. Research on this topic is ongoing, but it may be necessary to establish acceptable MRLs (even as marketing conditions) for off-target tissues if such tissues are to be continually tested for ractopamine concentrations, and particularly if they are tested for total ractopamine concentrations, in destination markets. In the meantime, livestock producers around the world, and specifically in the United States, are going to have to look for different ways to recover the losses incurred through the lost opportunity of exporting to countries with rigorous zero-tolerance ractopamine requirements for imports. They should also be cautious about cross-contamination and false positives that may occur as a consequence of other compound sources. Because the United States relies heavily on beef and pork exports, these marketing obstacles create tremendous barriers to trade. Implementation of current production technologies in the livestock industry leads to an additional $250 per head advantage over the animals not receiving growth promotants. This advantage is meaningless, however, if export markets are lost because of banned compounds. The countries involved in the current debate surrounding growth promotants need to find common ground so that proper nutrition can be delivered in a safe, economical, and sustainable manner.

## About the Authors

**Haley Davis** is a Ph.D. student in the Department of Animal Sciences at Colorado State University, and her research primarily focuses on food safety work within the Meat Science group. Haley is originally from Bakersfield, CA, where she was actively involved in FFA. She made the decision to attend CSU for her Bachelor’s in Animal Sciences and never looked back. Haley’s research includes validating various antimicrobials in commercial beef harvest facilities and microbiology labs, residue testing, and developing new antimicrobial application methods.

**Dr. K.E. (Keith) Belk** is a Professor and holds the Ken & Myra Monfort Chair in the Center for Meat Safety & Quality, Department of Animal Sciences at Colorado State University. He also is an adjunct Professor in the Colorado School of Public Health and has served as the consulting Director of Scientific Affairs for Food Safety Net Services, LLC since 2008. He has been employed by Safeway, Inc. as a beef buyer, and by the USDA-AMS in Washington, DC, as an International Marketing Specialist. He has authored or co-authored 220 refereed scientific journal articles addressing meat quality and safety, international marketing, and nutritional value of meat.
